# Serum-Based Assessment of Alopecia Areata Response to Treatment Using ATR-FTIR Spectroscopy

**DOI:** 10.3390/diagnostics15111369

**Published:** 2025-05-29

**Authors:** Charlotte Delrue, Arno Belpaire, Sigurd Delanghe, Matthijs Oyaert, Sander De Bruyne, Marijn M. Speeckaert, Reinhart Speeckaert

**Affiliations:** 1 Department of Nephrology, Ghent University Hospital, 9000 Ghent, Belgium; charlotte.delrue@ugent.be (C.D.); sigurd.delanghe@uzgent.be (S.D.); 2 Department of Dermatology, Ghent University Hospital, 9000 Ghent, Belgium; arno.belpaire@uzgent.be (A.B.); reinhart.speeckaert@uzgent.be (R.S.); 3 Department of Laboratory Medicine, Ghent University Hospital, 9000 Ghent, Belgium; matthijs.oyaert@uzgent.be; 4 Department of Diagnostic Sciences, Ghent University, 9000 Ghent, Belgium; sander.debruyne@jessazh.be; 5 Department of Laboratory Medicine, Jessa Ziekenhuis, 3500 Hasselt, Belgium; 6 Research Foundation-Flanders (FWO), 1000 Brussels, Belgium

**Keywords:** alopecia areata, ATR-FTIR spectroscopy, machine learning

## Abstract

**Background/Objectives**: Serum diagnostic tests for alopecia areata may be used to monitor response to treatment, aiding in the objective assessment of disease activity and helping to change treatment at an earlier point. Attenuated total reflection Fourier transform infrared (ATR-FTIR) spectroscopy offers a nondestructive and user-friendly approach for analyzing a wide range of samples. In this study, we evaluated whether ATR-FTIR spectroscopy combined with machine learning can detect alopecia areata and quantify disease activity. We also established whether patient-specific spectral differences correlate with response to therapy, offering molecular insight into treatment response. **Methods**: Serum samples from 42 patients with alopecia areata and 41 healthy donors were compared. Logistic regression models were developed to separate alopecia areata patients from controls and to monitor treatment response based on clinical scoring. **Results**: Significant spectral variations were found in the 3000–2800 cm^−1^ and 1800–1000 cm^−1^ regions corresponding to the principal biochemical constituents such as proteins, lipids, carbohydrates, and nucleic acids. The AUC of the logistic regression model for distinguishing alopecia areata patients from healthy controls was 0.85 (95% CI: 0.75–0.94) with a sensitivity of 0.89 and a specificity of 0.71. In terms of prediction of treatment response, the model showed discriminative potential (AUC = 0.86, 95% CI: 0.71–0.98), with distinct alterations in the spectrum, particularly in the Amide I band, associated with improvement in the patient’s condition. **Conclusions**: ATR-FTIR spectroscopy assisted by machine learning offers a serum-based solution for treatment monitoring in alopecia areata patients with clinical applicability. This technique has highly promising potential for the development of rapid, non-invasive, and objective biomarkers in autoimmune dermatology. Additional multi-center trials are required to validate and incorporate these spectral biomarkers into individual treatment regimens.

## 1. Introduction

Alopecia areata is a chronic, autoimmune disease with nonscarring hair loss, usually in the form of well-demarcated patches on the scalp but having the potential of extending to complete loss of scalp (alopecia totalis) or body hairs (alopecia universalis). The disease is present in approximately 1–2% of the population and can begin at any age [1,2]. The pathogenesis occurs through autoimmune assault on hair follicles, focusing on the immune privilege of the hair follicle. Alopecia areata is frequently observed in association with other autoimmune diseases, including thyroiditis, vitiligo, and rheumatoid arthritis, and emphasizes systemic dysregulation of immunity. Despite its clinical relevance, objective biomarkers for diagnosing alopecia areata or monitoring disease activity remain limited, and novel molecular approaches for measurement are required [3].

Serum diagnostic tests can detect early biochemical changes, help assess disease activity, and provide an objective measure of disease progression. They also hold promise in identifying biomarkers linked to the autoimmune processes underlying alopecia areata, such as specific antibodies or cytokines, which can assist in confirming diagnosis and offer insights into associated autoimmune conditions, such as Hashimoto’s thyroiditis or the underlying pathophysiology [4,5].

Attenuated total reflection Fourier transform infrared (ATR-FTIR) spectroscopy offers an innovative, noninvasive method for diagnosing alopecia areata by providing rapid molecular profiling of serum samples. This technique works by directing infrared light into the sample, where the light is partially absorbed by biochemical components such as proteins, lipids, and metabolites. The absorbed light creates a unique spectral fingerprint that represents the molecular composition of a sample. Given that ATR-FTIR spectroscopy uses an attenuated total reflection crystal, infrared light penetrates only a few micrometers into the sample, making it highly sensitive to surface-level biochemical changes. This makes it an ideal tool for detecting disease-specific alterations in serum composition [6,7]. FTIR spectroscopy resolves biochemically by measuring the characteristic vibrational frequencies of molecular bonds in biomolecules. Each functional group, whether made up of amide bonds in proteins, phosphate groups in nucleic acids, or C–H stretches in lipids, will absorb infrared radiation at a characteristic wavenumber. The absorbance pattern is a “molecular fingerprint” of the overall biochemical composition of the sample. Significantly, FTIR offers an indirect quantification of biochemical constituents through interaction with infrared light rather than concentration per se. This indirectness is highly sensitive to compositional and structural changes and enables recognition of subtle biochemical changes associated with mechanisms of disease. ATR-FTIR spectroscopy has also been shown to be promising in other dermatologic applications. It has been used to differentiate between healthy and psoriatic skin based on lipid and protein alterations in the stratum corneum, contributing to the assessment of disease severity [8,9]. It has also been used to monitor treatment responses in atopic dermatitis and to examine skin barrier dysfunction in terms of spectral properties related to hydration and lipid packing [10,11,12]. In cancer, ATR-FTIR has enabled non-invasive skin cancer examination of basal and squamous cell carcinomas by discriminating biochemical malignancy-associated signatures [13]. Combined, these applications underscore the usefulness of ATR-FTIR in dermatology for monitoring and diagnostic functions. Machine learning algorithms such as logistic regression can further enhance the diagnostic power of ATR-FTIR spectroscopy by processing complex spectral data and identifying subtle biochemical differences between patient groups. Logistic regression is a powerful and widely used machine-learning algorithm that is particularly well suited for binary classification problems. Its key strengths lie in its simplicity and interpretability. It outputs calibrated probabilities, which are particularly valuable when estimating risk or making threshold-based decisions, as in diagnostic models [14]. By coupling ATR-FTIR spectroscopy with machine learning, we can leverage these advanced analytical tools to improve the diagnostic accuracy and potentially assess disease activity.

The rationale for this study is the development of a reliable technique for monitoring the treatment response of alopecia areata. Current methods, although useful, are subjective and may not provide molecular-level insights into disease activity. ATR-FTIR spectroscopy, when combined with machine learning, offers a promising alternative by enabling the non-destructive and rapid analysis of serum samples. This approach has the potential to provide a deeper understanding of alopecia areata pathogenesis and facilitate the tracking of treatment response through biochemical markers. Specifically, we aimed to determine whether spectral differences in the serum of patients with alopecia areata could be identified and used to predict treatment response.

## 2. Materials and Methods

### 2.1. Study Population and Sample Collection

The study was approved by the ethics committee of Ghent University Hospital (BC-11730) and was conducted in accordance with the Declaration of Helsinki. Written informed consent was obtained from all participants involved in the study. Serum samples (*n* = 83) were collected from the Department of Dermatology at Ghent University Hospital, Belgium. These included 42 serum samples from individuals diagnosed with alopecia areata, confirmed by an expert dermatologist, and 41 control samples. Clinical metadata were available for all cases of alopecia areata. The severity of the affected zones was assessed using the Severity of Alopecia Tool II (SALT II) score [15]. These assessments were conducted at enrollment (time point 0, TP0), after 6 months (time point 1, TP1), and after 12 months (time point 2, TP2) for most patients. Serum samples were stored at −80 °C and thawed at room temperature (18–25 °C) for 15–20 min before analysis. A 0.5 µL aliquot of serum was applied directly to the internal reflection element (IRE) using a micropipette, and the drop was spread with the pipette tip to form a uniform thin film. The sample was allowed to dry for 30 s before spectral acquisition.

### 2.2. ATR-FTIR Spectral Acquisition and Preprocessing

**Figure 1:**
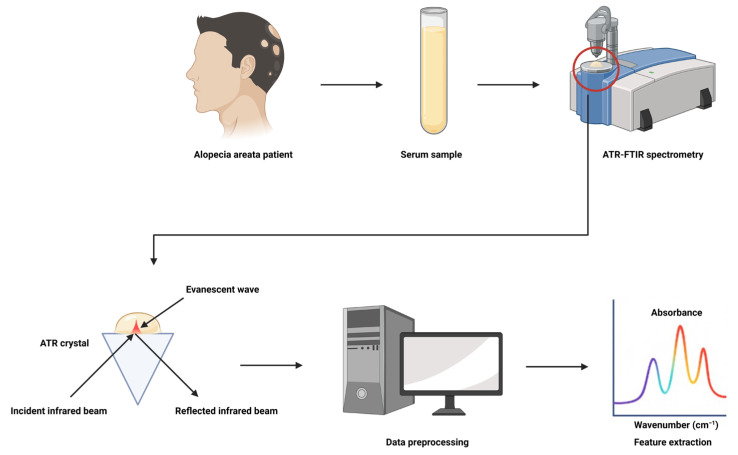
Schematic illustration of the ATR-FTIR spectroscopy procedure for serum analysis in alopecia areata. First, the serum is withdrawn from patients and placed on the ATR crystal of the FTIR spectrometer. The infrared radiation then enters the internal reflection element (IRE) and undergoes several internal reflections, forming an evanescent wave that penetrates a few microns into the serum sample. Next, biochemical components within the test sample absorb specific IR frequencies and produce an absorbance spectrum that is a molecular fingerprint. Finally, the spectral data are preprocessed and then fed into feature extraction followed by clinical interpretation.

ATR-FTIR spectra were obtained using a Perkin Elmer Spectrum Two spectrometer with a ZnSe crystal (2 × 2 mm) and Spectrum 10 software (Perkin Elmer, Waltham, MA, USA) (Figure 1). Spectra were acquired in the range of 4000–400 cm^−1^ at a resolution of 4 cm^−1^ with five co-added scans. Calibration was performed to ensure the accuracy of the spectral data for analysis. Only the spectral regions of interest (3000–2800 cm^−1^ and 1800–1000 cm^−1^) were retained in order to minimize noise from non-informative wavenumbers for the model of alopecia areata (*n* = 35) versus healthy controls (*n* = 41). For the model of responders (*n* = 17) and non-responders (*n* = 10), only the spectral region between 1750 and 1700 cm^−1^ was retained.

### 2.3. Statistical Analysis and Multivariate Data Analysis

Univariate data analysis was performed using Python (version 3.11.2) with the SciPy library (version 1.12.0). The Shapiro–Wilk test was used to assess data normality. Non-normally distributed data were reported as medians with interquartile ranges (IQR), and the Mann–Whitney U test was used for comparisons. Independent *t*-tests were used for normally distributed data, and the chi-squared test was used to assess the associations between categorical variables. A Bonferroni correction was applied to control for multiple comparisons, and a *p*-value of <0.05 was considered statistically significant. Data preprocessing, analysis, and visualizations were performed using Python with the libraries Pandas (version 2.2.2), SciPy (version 1.12.0), Scikit-learn (version 1.4.2), NumPy (version 1.26.4), Seaborn (version 0.13.2), and Matplotlib (version 3.8.4). To remove irrelevant scattered light and standardize the spectroscopic signals, several spectral filters were applied. First, normalization was performed using the SNV method. The spectra were converted to their first derivatives to enhance the resolution of the overlapping peaks. A Savitzky-Golay filter with 11 smoothing points was applied to reduce the noise and isolate important spectral features that could otherwise be obscured. Since the serum is a complex biological matrix, only the relevant spectral regions (3000–2800 cm^−1^ and 1800–1000 cm^−1^) were retained for analysis in order to minimize the influence of non-informative wavenumbers. Spectral data were analyzed using eight different machine learning models: support vector machines (SVM), K-nearest neighbors (KNN), linear discriminant analysis (LDA), PLS-DA, CatBoost classifier, logistic regression, extreme gradient boosting (XGBoost), and random forest. To assess and improve the robustness of the model, we employed LOOCV. In LOOCV, each observation is excluded one at a time, and the model is trained on the remaining data, with prediction errors aggregated to evaluate the performance. This process helped reduce overfitting and improve the generalizability of the model. The optimal classification threshold was determined using Youden index analysis of the receiver operating characteristic (ROC) curve. Model performance was evaluated using several key metrics, including sensitivity, specificity, positive predictive value (PPV), negative predictive value (NPV), and area under the curve (AUC), with 95% confidence intervals (CI) calculated using bootstrapping.

## 3. Results

### 3.1. Clinical Characteristics of the Study Population

The clinical characteristics of the study population are summarized in Table 1. The mean age of the patients with alopecia areata was 43.5 years (median: 46.5, interquartile range: 17.8), with 31.0% being men and 69.0% women. Familial alopecia areata was reported in 16.7% of the patients. The mean age at disease onset was 31.3 years (median, 30.5; interquartile range, 25.3). Among patients with alopecia areata, 40.4% had mild severity (0–20%), 26.2% had moderate severity (21–49%), and 31.0% had severe damage (≥50%), as measured by the SALT II score. Our cohort included different types of alopecia areata with 73.8% of patients being categorized as patchy, 21.4% totalis or universalis, one with alopecia areata barbae and one with diffuse hair loss (Table 1).

### 3.2. Spectral Differences Between Alopecia Areata and Controls

**Figure 2:**
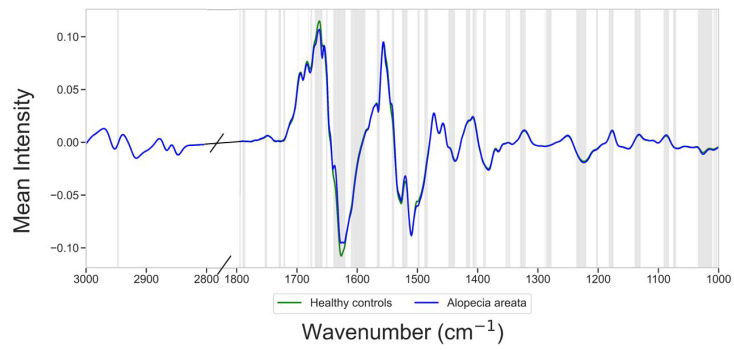
Mean pre-processed intensities of the wavenumbers in healthy controls (green line), which are significantly different from those in alopecia areata patients (blue line, highlighted with a grey shade).

**Figure 3:**
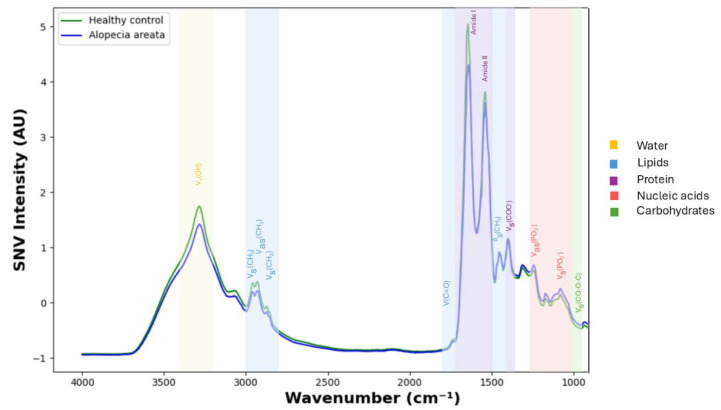
The average SNV-normalized absorbance spectra for alopecia areata patients (blue line) and control patients (green line) are shown, with emphasis on the median spectra from the full dataset. Key functional groups and their associated biomolecules are represented by specific wavenumbers in the mid-IR region. The lipid region is characterized by several distinct peaks, including C=O stretching of ester linkages at approximately 1740 cm^−1^, C-H stretching around 3000 cm^−1^ (saturated fats) and 3009 cm^−1^ (unsaturated fats), as well as C-H bending at approximately 1465 cm^−1^, which corresponds to methylene scissoring modes in fatty acid chains. The protein region is defined by Amide I bands, which correspond to C=O stretching, and Amide II bands, which represent N-H bending and C-N stretching. These bands are key markers of protein structure and provide insights into conformational changes in serum proteins. The nucleic acid region includes signals from the phosphate backbone of DNA and RNA, with PO_2_− asymmetric stretching occurring at approximately 1220 cm^−1^ and PO_2_− symmetric stretching at approximately 1080 cm^−1^. These peaks indicate nucleic acid integrity and may reflect disease-related changes in cellular turnover. Additionally, carbohydrates were identified by C-O stretching in the 1150–1000 cm^−1^ region, which highlights the presence of glycoconjugates and polysaccharides in the serum. Finally, the water bands absorbed in the 3550–3200 cm^−1^ region due to O-H stretching reflect the hydration states, which can influence the overall spectral profile.

During the exploration of the standard normal variate (SNV)-normalized first derivative spectra smoothed with a Savitzky–Golay filter (11 points), significant alterations were observed between healthy individuals and patients with alopecia areata (Figure 2). These alterations were primarily found in the region spanning 3000 cm^−1^ to 1000 cm^−1^, which corresponds to key biochemical constituents, including lipids, proteins, nucleic acids, and carbohydrates (all *p* < 0.05, Figure 3 and Table 2). By comparing the mean first-derivative spectra, distinct peaks were identified, especially in regions associated with protein absorption modes. One of the most significant peaks was found at 1661–1677 cm^−1^, which was influenced by proteins, mainly due to the C=O stretching vibration of peptides (Amide I). This peak is influenced by the secondary structure of proteins, providing insights into protein conformational changes in patients with alopecia areata. Within the Amide I band, multiple overlapping absorption peaks occur, each associated with specific secondary structural elements of the proteins. The 1654–1658 cm^−1^ region represents the α-helix presence, the 1624–1638 cm^−1^ region represents β-sheet formation, and the ~1666–1680 cm^−1^ region denotes β-turns of proteins. The Amide II band, located at 1520–1525, 1540–1542, 1563–1567, and 1587–1599 cm^−1^, is associated with in-plane C-N-H bending and C-N stretching in peptide bonds, reflecting alterations in the protein structure. Another peak linked to protein deformation was detected at 1563 cm^−1^, attributed to in-plane N–H bending and C–N stretching vibrations of the peptide bond, further supporting the hypothesis of altered protein metabolism in alopecia areata patients compared to healthy controls. Other notable differences were observed in the lipid and nucleic acid absorption modes, particularly in the CH_2_ bending band in the 1440–1448 cm^−1^ region. A peak at 1180 cm^−1^ was observed, corresponding to symmetrical PO_2_− stretching, which may indicate changes in the nucleic acid content of serum samples from patients with alopecia areata.

**Figure 4:**
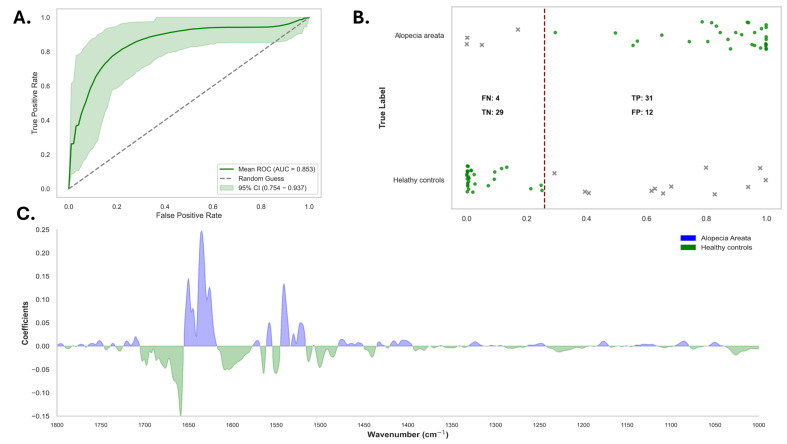
Performance of the logistic regression model in discriminating between patients with alopecia areata and healthy controls. (**A**) Receiver operating characteristic (ROC) curve of the logistic regression model. The curve plots the true positive rate (sensitivity) against the false positive rate (1-specificity) across all possible thresholds. The shaded region represents the 95% confidence interval (CI) of the mean ROC curve, reflecting the variability of the model performance across resampling iterations. The model achieved an area under the curve (AUC) of 0.853, with a 95% confidence interval (CI) ranging from 0.754 to 0.937. (**B**) Probability plot showing the predicted probabilities from the logistic regression model against true class labels. Each dot represents an individual participant, stratified by their true label: “alopecia areata” (top panel) or “healthy controls” (bottom panel). A classification threshold of 0.26 (indicated by the vertical dashed line) was applied to distinguish between the two classes. Dots to the right of the threshold are predicted as “Alopecia areata”, while those to the left are predicted as “healthy controls”. True positives (TP), true negatives (TN), false positives (FP), and false negatives (FN) are color-coded; black dots indicate correct classifications (TP and TN), whereas red crosses denote misclassifications (FP and FN). The model yielded 31 true positives and 29 true negatives, with 12 false positives and 4 false negatives. (**C**) Feature importance plot depicting the logistic regression coefficients across the vibrational spectral range (1700–100 cm^−1^). Each coefficient reflects the weight assigned to a specific wavenumber region during the model decision-making process. Positive coefficients (blue) indicate spectral regions that contribute more strongly to the prediction of “alopecia areata”, whereas negative coefficients (green) indicate spectral features associated with “healthy controls”. The spectral region around ~1650 cm^−1^ shows the highest positive contribution, likely corresponding to protein- or Amide-related bands, which may reflect disease-related biochemical alterations.

Serum, as a complex matrix, poses challenges for spectroscopic analysis because of the presence of many biochemical components. To minimize the influence of non-informative wavenumbers, only the relevant spectral regions [3000–2800 cm^−1^ (lipids) and 1800–1000 cm^−1^ (proteins and nucleic acids)] and only patients receiving no systemic treatment (*n* = 35) were retained for model construction. The logistic regression model was evaluated using LOOCV and demonstrated substantial efficacy in distinguishing healthy controls from patients with alopecia areata. The model achieved an AUC of 0.85 (95% CI: 0.75–0.94), indicating a discriminatory value at a classification threshold of 0.26 (Figure 4A).

At the optimal classification threshold, the logistic regression model demonstrated good overall performance. It achieved a sensitivity of 0.89 (95% CI: 0.79–0.94) and a specificity of 0.71 (95% CI: 0.60–0.80). Additionally, the model had a high NPV of 0.88 (95% CI: 0.79–0.93) and a PPV of 0.72 (95% CI: 0.61–0.81). The overall accuracy of the model was 0.79 (95% CI: 0.69–0.87), further indicating its satisfactory performance. The confusion matrix for the logistic regression model indicated that among the 41 true control cases, 29 were correctly classified as healthy, whereas 12 were misclassified as having alopecia areata. In contrast, out of the 35 true alopecia areata cases, the model accurately identified 31, with four misclassified as healthy (Figure 4B). In this cohort of four alopecia patients misclassified as controls by our model, all were women receiving local corticosteroid treatment with only their hair being affected, and none of them showed damage to the nails or the body. One was also affected by rheumatoid arthritis, which can affect this protein region in the infrared spectrum (Supplemental Table S1). These results reflect the model’s relatively high sensitivity, but highlight a moderate level of misclassification in controls.

In addition, a feature importance plot generated from the first-derivative spectra identified the most discriminative spectral regions for the model. These regions primarily corresponded to biochemical components such as proteins (1650–1600 cm^−1^ and 1550–1400 cm^−1^), lipids (1448–1440 cm^−1^), and nucleic acids (1250–1200 cm^−1^) (Figure 4C). These findings suggest that changes in these biochemical components are key to distinguishing alopecia areata patients from healthy controls.

**Figure 5:**
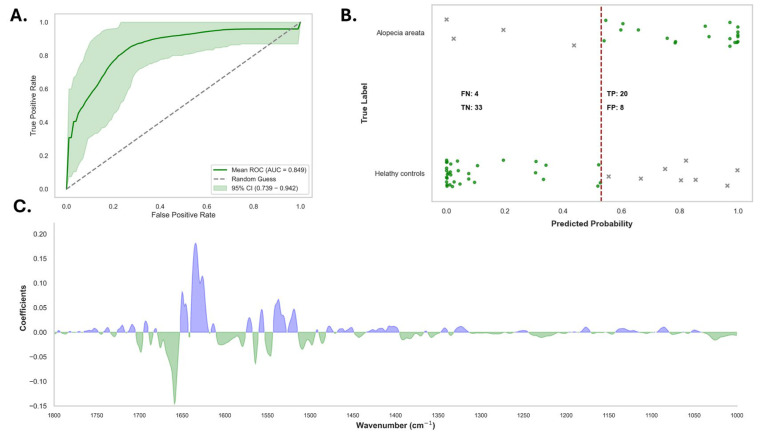
Performance of the logistic regression model for discriminating between patients with alopecia areata without IMID and healthy controls. (**A**) Receiver operating characteristic (ROC) curve of the logistic regression model. The curve plots the true positive rate (sensitivity) against the false positive rate (1-specificity) across all possible thresholds. The shaded region represents the 95% confidence interval (CI) of the mean ROC curve, reflecting the variability of the model performance across resampling iterations. The model achieved an area under the curve (AUC) of 0.849, with a 95% confidence interval (CI) ranging from 0.739 to 0.942. (**B**) Probability plot showing the predicted probabilities from the logistic regression model against true class labels. Each dot represents an individual participant, stratified by their true label: “alopecia areata” (top panel) or “healthy controls” (bottom panel). A classification threshold of 0.53 (indicated by the vertical dashed line) was applied to distinguish between the two classes. Dots to the right of the threshold are predicted as “alopecia areata”, while those to the left are predicted as “healthy controls”. True positives (TP), true negatives (TN), false positives (FP), and false negatives (FN) are color-coded; black dots indicate correct classifications (TP and TN), whereas red crosses denote misclassifications (FP and FN). The model yielded 33 true positives and 20 true negatives, with 8 false positives and 4 false negatives. (**C**) Feature importance plot depicting the logistic regression coefficients across the vibrational spectral range (1700–100 cm^−1^). Each coefficient reflects the weight assigned to a specific wavenumber region during the model decision-making process. Positive coefficients (blue) indicate spectral regions that contribute more strongly to the prediction of “alopecia areata,” whereas negative coefficients (green) indicate spectral features associated with “healthy controls”. The spectral region around ~1650 cm^−1^ shows the highest positive contribution, likely corresponding to protein- or Amide-related bands, which may reflect disease-related biochemical alterations.

In the next step, we excluded all alopecia areata patients affected by IMID comorbidities. At the classification threshold of 0.55, the logistic regression model confirmed good overall performance (Figure 5A). It achieved a sensitivity of 0.84 (95% CI: 0.69–0.96) and a specificity of 0.73 (95% CI: 0.59–0.86). Additionally, the model had a high NPV of 0.88 (95% CI: 0.76–0.97) and a PPV of 0.65 (95% CI: 0.48–0.82). The overall accuracy of the model was 0.77 (95% CI: 0.68–0.86), further indicating its satisfactory performance. The confusion matrix for the logistic regression model indicated that among the 41 true control cases, 33 were correctly classified as healthy, whereas 8 were misclassified as having alopecia areata. In contrast, out of the 24 true alopecia areata cases, the model accurately identified 20, with four misclassified as healthy (Figure 5B). In this cohort of four alopecia patients misclassified as controls by our model, three were women with patchy alopecia areata receiving local corticosteroid treatment with only affected hair, and none of them showed damage to the nails or the body. The other one was a man affected by alopecia areata totalis. As aforementioned, the feature importance plot generated from the first-derivative spectra identified the most discriminative spectral regions for the model. These regions also primarily corresponded to biochemical components such as proteins (1650–1600 cm^−1^ and 1550–1400 cm^−1^), lipids (1448–1440 cm^−1^), and nucleic acids (1250–1200 cm^−1^) (Figure 5C).

### 3.3. Responders Versus Non-Responders in Alopecia Areata Patients

**Figure 6:**
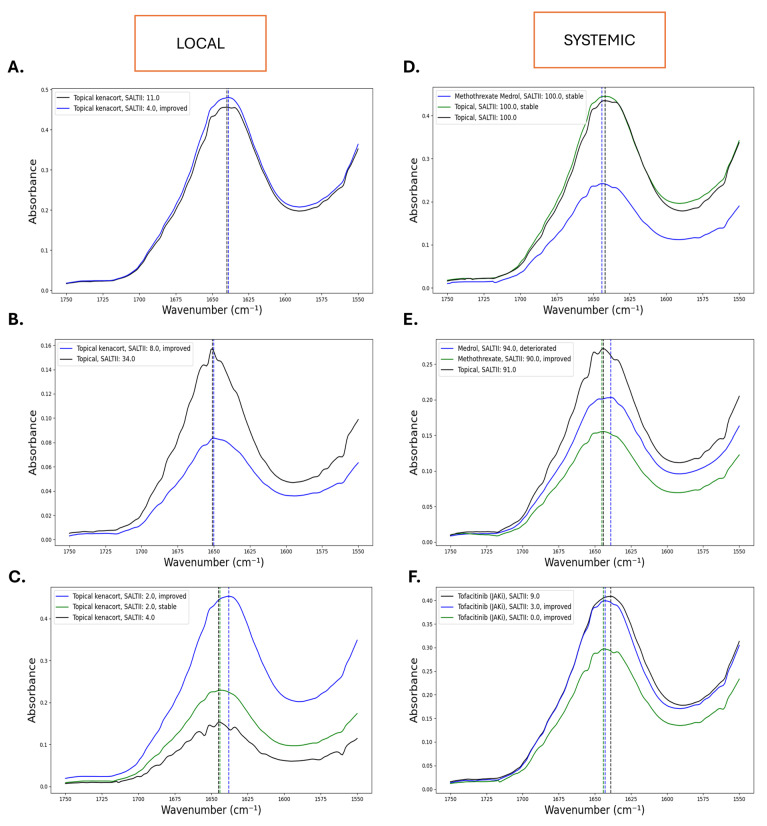
Fourier transform infrared (FTIR) spectroscopy absorbance spectra in the Amide I band region (1750–1550 cm^−1^) of six patients’ serum samples (Panels (**A**–**F**)) with different treatments for alopecia. Each panel shows FTIR spectra measured at three time points: TP0 (black line), TP1 (blue line), and TP2 (green line). The *y*-axis is the absorbance level, and the *x*-axis is the wavenumber (cm^−1^). Dashed vertical lines are used to mark the position of the peak in the Amide I region, which reflects the protein structural features. Labeling on each panel shows the treatment received at each time point, the corresponding Severity of Alopecia Tool (SALT) score, and the response (e.g., improved, stable, or worsened). Therapies include JAK inhibitors such as tofacitinib and baricitinib, immunosuppressants including Methotrexate and Medrol, and other medications including intralesional triamcinolone and diphencyprone. Changes in the spectrum over time, particularly in position and peak intensity, reflect protein conformational changes that may be associated with the therapeutic effect.

In the next phase of the study, we aimed to assess the treatment response in patients with alopecia areata using a logistic regression model to differentiate between responders (*n* = 17) and non-responders (*n* = 10). Follow-up data of 27 patients were available, and we included patients with alopecia areata who received local or systemic treatment. By comparing the mean first-derivative spectra, distinct significant wavenumbers were identified, especially in the Amide I peak. The most significant wavenumbers were 1747–1743 cm^−1^, 1728–1720 cm^−1^, 1716–1711 cm^−1^, and 1710–1709 cm^−1^, which are associated with secondary protein formation. In addition to the significant difference in absorbance intensity, there was also a shift in the Amide I band observed in responders compared to that in non-responders. Figure 6 shows the mean infrared spectra of six patients, each followed at baseline, 6 months, and 12 months, highlighting spectral evolution in the Amide I band in relation to clinical outcomes. In Panel A, which represents a responder on local corticosteroids, the spectrum shifts to the right side (lower wavenumbers) of the IR spectrum after 6 months, indicating clinical improvement. Panel B depicts a patient treated with topical corticosteroids; at 6 months, the patient improved clinically, resulting in a right shift of the IR spectrum. In Panel C, the spectrum shows an almost stable position of the Amide I peak at 6 months corresponding to clinical stability. At 12 months, there is a clinical improvement resulting in a right shift of the Amide I peak.

Panel D presents a patient receiving Methothrexate and Medrol who remained stable during follow-up; however, IR spectra faced interference from systemic treatment. In Panel E, the patient also received Medrol and deteriorated after 6 months; however, the Amide I peak shifted to the right side of the IR spectrum. Contradictorily, after 12 months, the patient was treated with Methothrexate, and the Amide I peak shifted to the left side as the patient improved. Finally, Panel F displays a patient being treated with JAK inhibitors, and it showed a shift to the left side of the Amide I peak as it improved clinically, which is also contrary to the effects seen in locally treated patients.

Generally, shifts in the Amide I band appear to correlate with treatment response, with right shifts often associated with clinical improvement in locally treated patients. However, these effects may be masked by receiving systemic treatment.

**Figure 7:**
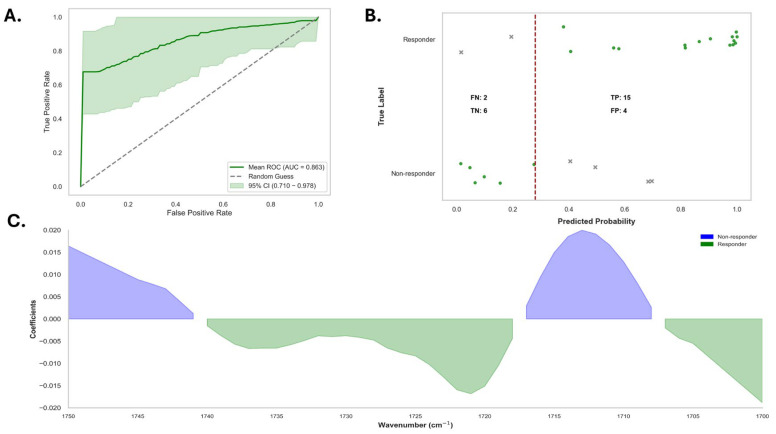
Model performance and importance of spectral features for the classification of alopecia areata. (**A**) Receiver operating characteristic (ROC) curve illustrating the classification performance of the model, with a mean area under the curve (AUC) of 0.863 and a shaded area representing the 95% confidence interval. The broken line represents the random classifier performance. (**B**) Predicted probability plot with classification outputs, actual labels for alopecia areata, and healthy controls divided by a decision boundary (red dashed line), with counts for true positives (TP), false positives (FP), true negatives (TN), and false negatives (FN). (**C**) Visualization of the importance of spectral features as model coefficients against wavenumbers (cm^−1^). Contributions to classification are shown as positive and negative for responders (green) and non-responders (blue), respectively, and where important spectral regions between groups are distinguished.

Further, the logistic regression model yielded a leave-one-out cross-validated AUC of 0.86 (95% CI: 0.71–0.98) (Figure 7). At a classification threshold of 0.28, the model’s performance was robust with a sensitivity of 0.88 (95% CI: 0.71–0.96), though specificity was lower at 0.50 (95% CI: 0.32–0.68). The PPV and NPV were similar at 0.75 (95% CI: 0.56–0.86) and 0.71 (95% CI: 0.53–0.85), respectively. Overall, the model achieved an accuracy of 0.74 (95% CI: 0.55–0.87).

The confusion matrix revealed that among the patients classified as responders (*n* = 17), 15 were correctly identified, whereas two responders were misclassified in this group. In contrast, among the 10 non-responders, 6 were correctly classified and 4 cases were misidentified as non-responders. In the cohort of four ‘misclassified’ non-responders, three had at least one immune-mediated inflammatory disease (e.g., autoimmune thyroid disease, psoriasis). The other patient was a man with alopecia areata totalis who was treated with a JAK inhibitor. The false-negative cases included two women with atopic dermatitis. One patient was affected by autoimmune thyroid disease and was treated with a JAK inhibitor (Supplemental Tables S1 and S2).

## 4. Discussion

This study demonstrates that ATR-FTIR spectroscopy in combination with logistic regression modelling can differentiate between serum samples from patients with alopecia areata and healthy controls as well as provide early information on treatment responsiveness. Our work adds to the growing body of literature supporting the use of infrared-based diagnostic tools in autoimmune and inflammatory diseases, offering both molecular sensitivity and clinical interpretability.

Experimentally reported differences in spectra of alopecia areata patients and controls, particularly in the 3000–2800 cm^−1^ [16] and 1800–1000 cm^−1^ [17,18] portions of the spectrum, are associated with changes in the simple biochemistry components in the serum. These regions are associated with vibrational modes of proteins, nucleic acids, lipids, and carbohydrates, which are commonly dysregulated in autoimmune diseases. Specifically, Amide I (~1650 cm^−1^) band shifts reflect the protein secondary structure and conformational changes [19,20]. These findings complement those of previous studies using infrared spectroscopy to distinguish disease-specific protein folding and aggregating patterns in autoimmune diseases. Identification of peaks in the 1440–1448 cm^−1^ band (lipid CH_2_ bending) and at 1180 cm^−1^ (symmetric PO_2_^−^ stretching of nucleic acids) validates the speculation that autoimmune activity in alopecia areata could occur through systemic metabolic alteration. These must be immune-mediated inflammatory alterations, oxidative stress, or dyslipidemia, which are characteristics of autoimmune pathogenesis such as alopecia areata [4].

The model derived from diagnostics yielded an AUC of 0.86 with high sensitivity (0.89) and specificity (0.71) and with high potential for clinical application in alopecia areata screening. This is consistent with the findings of earlier research using FTIR for the serum-based discrimination of cancer and metabolic diseases, where similar AUC values (~0.85–0.95) have been reported [7]. Interestingly, the feature importance plot revealed that the discriminatory power was largely from protein-associated regions [16,21,22], as would be anticipated considering the well-characterized immune proteomic abnormalities of alopecia areata. Although four patients with alopecia areata were falsely classified as controls, the medical files showed that these individuals were on local corticosteroid therapy and had no nail or body involvement, which might have led to less severe biochemical alterations. They also suffered from rheumatoid arthritis, a known confounder due to overlapping inflammatory protein patterns that affect the spectral interpretation.

The ATR-FTIR spectroscopic analysis also differentiated responders from non-responders. In particular, the carbonyl (C=O) stretching region showed a consistent leftward shift in responders, which is consistent with the structural normalization of serum proteins. This suggests that FTIR-based assessment could offer a tool to monitor molecular-level changes correlating with clinical improvements and an objective complement to subjective measures such as the SALT score. The AUC of the model for treatment response classification was 0.85 with high sensitivity (0.88) but low specificity (0.50), indicating possible clinical monitoring at the expense of false positives. Most misclassified patients had immune-mediated comorbid diseases (e.g., autoimmune thyroiditis and psoriasis), indicating that spectral biomarkers require further optimization to rule out alopecia areata-specific signatures. These results agree with the growing number of publications in favor of the ability of serum spectroscopy to stratify immunological diseases [7,21,23,24]. In psoriasis, hyperproliferation of keratinocytes, altered lipid metabolism in the skin, and inflammatory infiltration might enhance protein- and lipid-associated bands, especially Amide I/II and CH-stretching regions [8]. In autoimmune thyroid disease, systemic immune activation, antibody production (e.g., anti-TPO), and increased oxidative stress may elevate bands linked to proteins (immunoglobulins) and nucleic acids (cell death, turnover). Treatment with JAK inhibitors typically results in differences in the spectral regions 3100–3000 cm^−1^ (aromatic C–H), 1650–1500 cm^−1^ (C=C and C=N- stretching, ring vibrations, particularly pyrimidines), 1450–1350 cm^−1^ (CH_3_, CH_2_− bending), 1300–1100 cm^−1^ (C–N, C–O vibrations), and 1000–600 cm^−1^ (aromatic ring deformation, out-of-plane, very characteristic for the fingerprint of this molecule) [22]. From the clinician’s perspective, ATR-FTIR spectroscopy is a valuable potential swift, non-invasive, and cost-effective tool to contribute towards the measurement of response to treatment for alopecia areata. Differing from traditional visual scoring systems that are subjective in nature, this serum-based method offers objective biochemical data that may arise prior to visible clinical change. Doctors can use this device to identify non-responders early and introduce augmented treatment or therapy switching earlier, potentially improving the results of patients. Additionally, because the test requires only a tiny amount of serum and no advanced processing, it can easily be integrated into routine dermatology practice or lab protocols. Standard spectral cutpoints can, in the future, guide precision medicine approaches to customize treatments based on biochemical profiles and prevent unnecessary systemic immunosuppression.

The limitations of this study are as follows: Due to the small sample size and the presence in the population of topically and systemically treated patients, the results can be heterogeneous. Caution needs to be taken by interpreting the performance metrics calculated on a limited dataset. Second, as FTIR offers high biochemical resolution, the methodology is still open to external confounders, such as drugs, comorbidities, and storage conditions. Third, the comparatively low specificity of the model for treatment response indicates the need for improved spectral preprocessing and model calibration.

Our findings should be interpreted as preliminary and hypothesis-generating, pending validation in larger multicenter studies. Future research is needed to enroll larger multicenter disease subtype- and treatment-stratified cohorts. Standardization of spectral acquisition, preprocessing, and interpretation is necessary for clinical reliability. Subsequent studies must compare ATR-FTIR-based diagnosis with existing clinical practice and assess cost-effectiveness and ease of introduction in dermatologic clinics. The combination of longitudinal spectrum monitoring with proteomic or metabolomic data may improve spectroscopic biomarker interpretability and stability. Multimodal models incorporating other serum tests (e.g., autoantibody and cytokine levels) could potentially improve the discriminatory performance and enable an integrated biomarker strategy. It is only with such confirmation that this promising technique can be a trusty instrument in precision dermatology.

## 5. Conclusions

This study provides strong preliminary evidence that ATR-FTIR spectroscopy, when combined with machine learning, can serve as a noninvasive, rapid, and informative tool for both diagnosis and treatment monitoring in alopecia areata. Biochemical signatures captured in serum reflect the underlying pathophysiological processes and offer a molecular window for disease activity. With further validation, this technique may enhance personalized treatment strategies and improve clinical outcomes in alopecia areata.

## Figures and Tables

**Table 1:** Demographic and clinical characteristics of alopecia areata patients.

Clinical Characteristics	Value
Men, *n*/total (%)	13/42 (31.0%)
Women, *n*/total (%)	29/42 (69.0%)
Age (years), mean (median, IQR)	43.5 (46.5, 17.8)
Age of onset AA (years), mean (median, IQR)	31.3 (30.5, 25.3)
Familial AA	
Yes, *n*/total (%)	7/42 (16.7%)
No, *n*/total (%)	35/42 (83.3%)
SALT II score at TP0 (%), mean (median, IQR)	42.1 (29.0, 82.0)
BMI (kg/m^2^), mean (median, IQR)	25.8 (24.7, 6.0)
Type of AA	
Patchy, *n*/total (%)	31/42 (73.8%)
Totalis or universalis, *n*/total (%)	9/42 (21.4%)
Barbae, *n*/total (%)	1 (2.4%)
Diffuse, *n*/total (%)	1 (2.4%)
IMID	
No, *n*/total (%)	29/42 (69.0%)
Yes, *n*/total (%)	13/42 (31.0%)
Inflammatory bowel disease	1/42
Psoriasis	2/42
Rheumatoid arthritis	2/42
Systemic lupus erythematosus	1/42
Thyroid disease	5/42
Microscopic polyangiitis	1/42
Sapho syndrome	1/42
Immune thrombotic thrombocytopenic purpura	1/42
Vitiligo	1/42
Addison’s disease	1/42
Sarcoidosis	1/42
APECED syndrome	1/42
Therapy at TP0	
None	12/42 (28.5%)
Topical corticosteroids	23/42 (54.8%)
Topical DCP	1 (2.4%)
Systemic treatment	6/42 (14.3%)
Methotrexate	3/6 (50.0%)
JAK inhibitors	2/6 (33.3%)
Cyclosporine	1/6 (16.7%)

Abbreviations: IQR, interquartile range; BMI, body mass index; IMID, immune-mediated inflammatory disease; APECED, autoimmune polyendocrinopathy-candidiasis-ectodermal dystrophy; DCP, diphenylcyclopropenone; JAK, Janus kinase.

**Table 2:** Overview of vibrational bands and their corresponding wavenumber ranges (cm^−1^) linked to biomolecules.

Vibrational Mode	Typical Wavenumber Range (cm^−1^)	Significant Wavenumber Between Alopecia Areata Patients and Healthy Controls (cm^−1^)
CH_3_ stretching (symmetric)	~2870–2960	2946–2948
Carbonyl (C=O) stretching	~1700–1760	1720–1730, 1750–1753
Amide I	~1600–1700	1661–1677, 1681, 1698
Amide II	~1500–1580	1520–1525, 1540–1542, 1563–1567, 1587–1599
CH_2_ bending	~1430–1470	1440–1448
Symmetric COO^−^ stretching	~1300–1420	1347–1353, 1387–1390, 1402, 1404–1408, 1413–1419
Symmetric and asymmetric PO_2_^−^ stretching	~1080–1250	1130–1139, 1175–1182, 1201–1203, 1220–1236

## Data Availability

The data that support the findings of this study are available from the corresponding author upon reasonable request.

## Supplementary Materials

The following supporting information can be downloaded at: https://www.mdpi.com/article/10.3390/diagnostics15111369/s1, Table S1: Misclassification table of the false negatives in the model of alopecia areata patients versus healthy controls; Table S2: Misclassification table of the false positives in the model of responders versus non-responders; Table S3: Misclassification table of the false negatives in the model of responders versus non-responders.

## Abbreviations

AA	Alopecia Areata
ATR-FTIR	Attenuated Total Reflection-Fourier Transform Infrared
AUC	Area Under the Curve
BMI	Body Mass Index
CI	Confidence Interval
DCP	Diphenylcyclopropenone
FTIR	Fourier Transform Infrared
IMID	Immune-Mediated Inflammatory Disease
IRE	Internal Reflection Element
IQR	Interquartile Range
JAK	Janus Kinase (inhibitor)
LOOCV	Leave-One-Out Cross-Validation
NPV	Negative Predictive Value
PPV	Positive Predictive Value
ROC	Receiver Operating Characteristic
SALT II	Severity of Alopecia Tool II
SNV	Standard Normal Variate
