# Supplementary material for: Serum-Based Assessment of Alopecia Areata Response to Treatment Using ATR-FTIR Spectroscopy
Source: Diagnostics (Basel). 2025 May 29;15(11):1369. doi: 10.3390/diagnostics15111369 (PMC12155509; doi:10.3390/diagnostics15111369)
Supplement: Supplementary file 1 [file diagnostics-15-01369-s001.zip › diagnostics-3637686-supplementary.pdf]

diagnostics

MDPI

Supplemental Table S1. Misclassification table of the false negatives in the model of alopecia areata patients versus healthy controls.

Predicted_Probability	Salt II score	Age (years)	Sex	BMI (kg/m²)	IMID	Type of AA	Age of onset AA	Therapy	SBN hair	SBN body	SBN nails	Atopy
0.170002	4.0	48	F	32.2	None	Patchy	32.0	Topical kenacort	S1	B0	N0	Yes
0.000648	9.0	46	F	23.1	None	Patchy	40.0	None	S1	B0	N0	No
0.000349	34.0	59	F	27.8	None	Patchy	57.0	Topical corticosteroids	S2	B0	N0	Yes
0.050115	32.0	49	F	23.0	RA	Patchy	Unknown	Topical corticosteroids	S2	B0	N0	No

Abbreviations: SALT II, severity of alopecia tool; IMID, immune-mediated inflammatory disease; RA, rheumatoid arthritis; AA, alopecia areata; SBN, scalp, body, and nail classification.

Supplemental Table S2. Misclassification table of the false positives in the model of responders versus non-responders.

Predicted_Probability	TP (months)	Age (years)	Sex	BMI (kg/m^2)	IMID	Family AA	Type of AA	Age of onset AA (years)	Therapy	Salt II	SBN hair	SBN body	SBN nails	Atopy	Remarks
0.405728	6	47	F	19.3	Thyroid disease	No	Patchy	44	Topical kenacort	4	S1	B0	N0	No	
0.495196	6	59	F	24.3	Microscopic polyangiitis	No	Diffuse	30	Methotrexate	0	S1	B0	N0	No	diffuse AA
0.695862	12	49	F	24.9	Psoriasis	No	Patchy	18	Topicalkenacort	10	S1	B0	N0	No	
0.684062	6	49	M	20.4	None	No	Totalis or universalis	44	Upadacitinib (JAKi)	100	S5	B2	N1	No	

Abbreviations: TP, timepoint; BMI, body mass index; SALT II, severity of alopecia tool; IMID, immune-mediated inflammatory disease; RA, rheumatoid arthritis; AA, alopecia areata; SBN, scalp, body, and nail classification; JAKi, Janus kinase inhibitor.

Supplemental Table S3. Misclassification table of the false negatives in the model of responders versus non-responders.

Predicted_Probability	TP (months)	Age (years)	Sex	BMI (kg/m²)	IMID	Family AA	Type of AA	Age of onset AA (years)	Therapy	Salt II	SBN hair	SBN body	SBN nails	Atopy
0.015744	6	48	F	32.2	No	No	Patchy	32	Topical kenacort	2	S1	B0	N0	Yes
0.195026	12	51	F	22.6	Thyroid disease	No	Patchy	47	Olumiant (JAKi)	0	S4B	B2	N0	Yes

Abbreviations: TP, timepoint; BMI, body mass index; SALT II, severity of alopecia tool; IMID, immune-mediated inflammatory disease; RA, rheumatoid arthritis; AA, alopecia areata; SBN, scalp, body, and nail classification; JAKi, Janus kinase inhibitor.
